# Supplementary material for: Correlation-driven transport asymmetries through coupled spins in a tunnel junction
Source: Nat Commun. 2017 Jan 11;8:14119. doi: 10.1038/ncomms14119 (PMC5260857; doi:10.1038/ncomms14119)
Supplement: Supplementary Information — Supplementary Figures and Supplementary References. [file ncomms14119-s1.pdf]

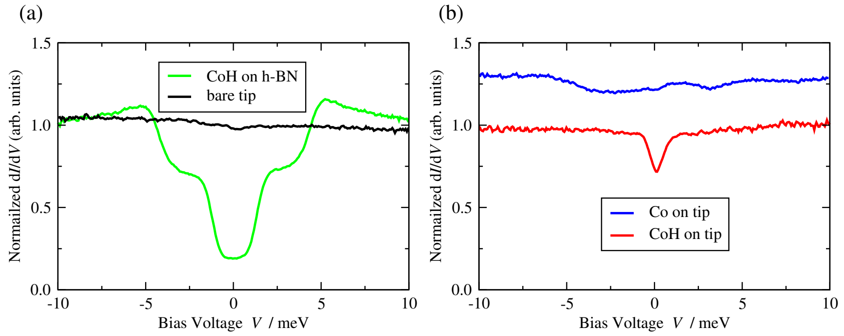

Supplementary Figure 1: **Spectroscopy of bare and Co-functionalized tips**. **a,** Typical spectrum of a bare Pt tip measured on the h-BN/Rh(111) sample (black). For comparison a spectrum obtained on a ($S = 1$) CoH complex is shown (green). **b,** Compared to the flat spectrum of the bare tip the spectrum of the Co-functionalized tip from Fig. 1d of the main paper shows some small modulations in the dI/dV signal when measured on h-BN/Rh(111) (blue). In rare occasions functionalization of the tip apex leads to an attached CoH complex. These tips show a dip of approx. 0.4 mV half-width at half-maximum around zero bias. The spectra of the functionalized tips are similar as data obtained on Co and CoH adsorbed on a Pt(111) surface[1].

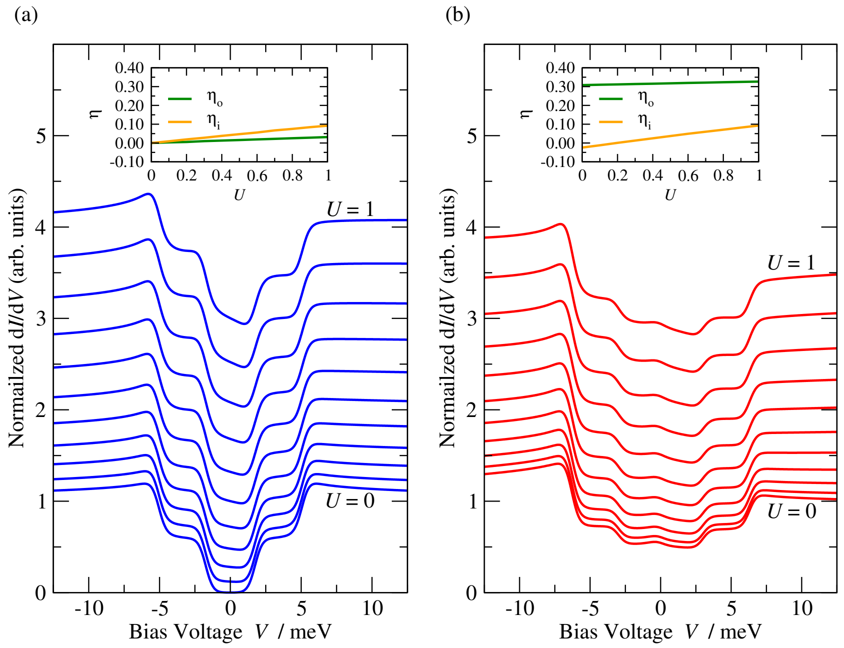

Supplementary Figure 2: **Influence of potential scattering on the spectra.** Simulated spectra for the lowest and highest experimentally measured conductance, $G_s = 33$ nS (**a,** blue) and $G_s = 630$ nS (**b,** red), of the dataset shown in Figure 1g of the main text, with different potential scattering terms ranging from $U = 0$ to $U = 1$. Spectra are offset vertically for better visualization. Insets show the dependence of the step asymmetry $\eta_{i,o}$ on U. While the asymmetry of the outer step η_o is only weakly influenced, the η_i reaches with increasing U up to 10%.

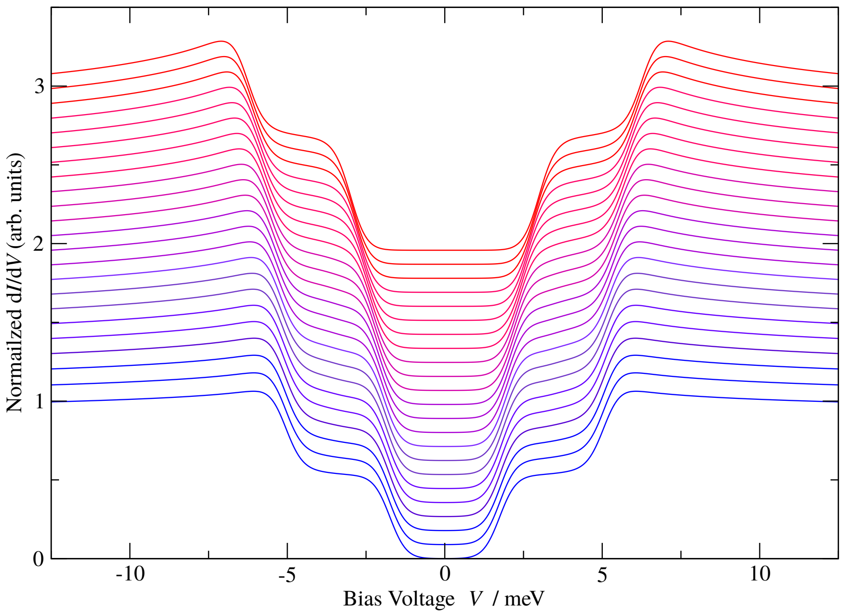

Supplementary Figure 3: **Simulation assuming change in magnetic anisotropy.** Simulated spectra assuming that the approaching tip (bottom (blue): low, top (red): high conductance) only changes the anisotropy parameters D and E to match the observed step positions (Figure 1g of the main text) of the experimental data. We see that these spectra neither match the observed step asymmetries nor show the intensity reduction of the energetically lower steps at higher conductance. Spectra are offset vertically for better visualization.

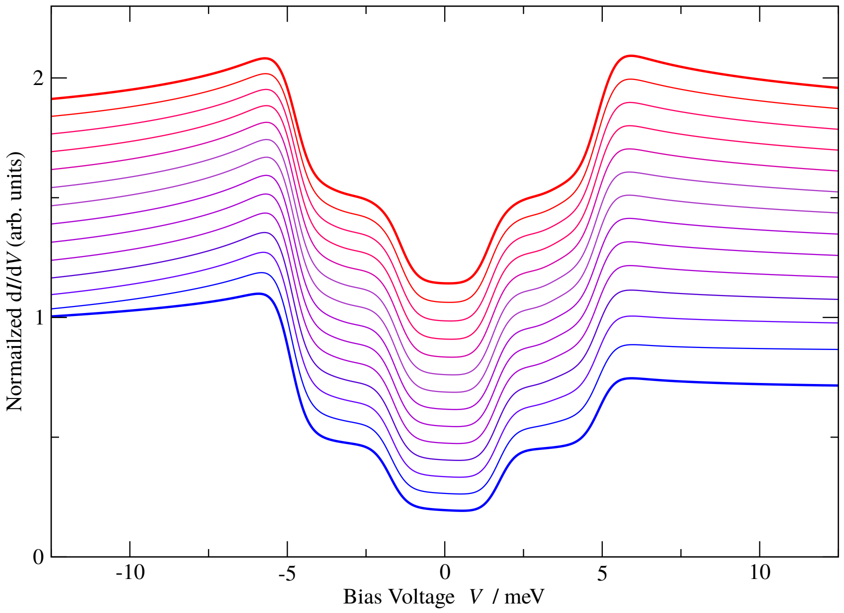

Supplementary Figure 4: **Simulation of spectra in magnetic field**. Simulation reproducing the data shown in figure 4 of the main text in an external field of $B_z = 5$ T. Spectra are offset vertically for better visualization.

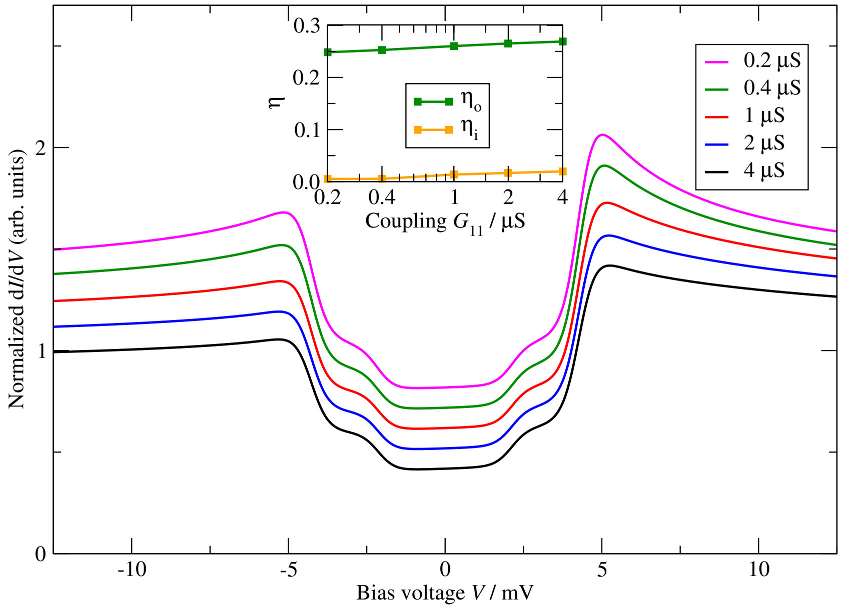

Supplementary Figure 5: **Influence of the coupling to the sample**. Simulated spectra for the highest experimentally measured conductance, $G_s = 300$ nS, of the dataset shown in Figure 1h of the main text, with different couplings of the S_1 spin to the sample substrate electrons. Spectra are offset vertically for better visualization. Insets show the dependence on the step asymmetries $\eta_{\mathrm{i,o}}$ which are only marginally influenced.

Supplementary References:

[1] Q. Dubout, F. Donati, C. Wäckerlin, F. Calleja, M. Etzkorn, A. Lehnert, L. Claude, P. Gambadella, and H. Brune, Phys. Rev. Lett. **114**, 106807 (2015).
